# Development and External Validation of Integrated Machine Learning-Based Prognostic Model in Oropharyngeal Head and Neck Cancer Using the Systemic Inflammatory Response Index

**DOI:** 10.3390/cancers17233820

**Published:** 2025-11-28

**Authors:** Anurag K. Singh, Sung Jun Ma, Dukagjin Blakaj, Simeng Zhu, Neil D. Almeida, Andrew Koempel, Guangwei Yuan, Grace Wang, Kimberly Wooten, Vishal Gupta, Ryan McSpadden, Moni A. Kuriakose, Michael R. Markiewicz, Song Yao, Wesley L. Hicks, Mukund Seshadri, Elizabeth A. Repasky, Elizabeth G. Bouchard, Mark K. Farrugia, Han Yu

**Affiliations:** 1 Department of Radiation Medicine, Roswell Park Comprehensive Cancer Center, Elm and Carlton Streets, Buffalo, NY 14203, USA; 2 Department of Radiation Oncology, The Arthur G. James Cancer Hospital and Richard J. Solove Research Institute, The Ohio State University Comprehensive Cancer Center, 460 West 10th Avenue, Columbus, OH 43210, USA; 3 Department of Biostatistics and Bioinformatics, Roswell Park Comprehensive Cancer Center, Elm and Carlton Streets, Buffalo, NY 14203, USAgrwang345@gmail.com (G.W.); 4 Department of Head and Neck Surgery, Roswell Park Comprehensive Cancer Center, Elm and Carlton Streets, Buffalo, NY 14203, USA; kimberly.wooten@roswellpark.org (K.W.); vishal.gupta@roswellpark.org (V.G.);; 5 Department of Oral and Maxillofacial Surgery, School of Dental Medicine, University at Buffalo, The State University of New York, 3435 Main Street, Buffalo, NY 14214, USA; 6 Department of Neurosurgery, Department of Surgery, Jacobs School of Medicine and Biomedical Sciences, University at Buffalo, The State University of New York, 955 Main Street, Buffalo, NY 14203, USA; 7 Department of Cancer Prevention and Control, Roswell Park Comprehensive Cancer Center, Elm and Carlton Streets, Buffalo, NY 14203, USA; 8 Department of Oral Oncology, Roswell Park Comprehensive Cancer Center, Elm and Carlton Streets, Buffalo, NY 14203, USA; 9 Department of Immunology, Roswell Park Comprehensive Cancer Center, Elm and Carlton Streets, Buffalo, NY 14203, USA

**Keywords:** oropharynx, neutrophil, lymphocyte, monocyte, squamous cell carcinoma

## Abstract

**Importance:** Patient with head and neck cancer of the oropharynx (HNC-OROP) undergo curative-intent definitive or post-operative radiation therapy. The systemic inflammation response index (SIRI) has independent prognostic capacity in HNC-OROP. We hypothesized that the use of SIRI may produce a parsimonious model of HNC-OROP outcomes. **Objective:** We aimed to investigate the prognostic utility of systemic inflammatory response index (SIRI) in oropharyngeal head and neck cancer patients who underwent radiation therapy. Design, Setting, and Participants: Random survival forest (RSF) machine learning was used to model survival in 568 oropharyngeal cancer patients in this retrospective cohort study. SIRI was calculated via pre-treatment bloodwork. Model validation was performed in an external cohort of 421 oropharyngeal cancer patients. **Exposures:** Exposure was curative-intent definitive or post-operative radiation therapy for head and neck cancer of the oropharynx (HNC-OROP). **Results:** This is a retrospective study with 568 and 421 patients in the Roswell Park and external Ohio State University cohorts. We evaluated full and reduced RSF models and a robust decision tree model. The C-index of the models was 0.758 (RSF full), 0.725 (RSF reduced), and 0.702 (decision tree). The incorporation of SIRI (with performance status and smoking history) into a machine learning model identified three risk-groups that significantly stratified overall survival (*p* < 0.0001). These findings were validated in the external validation cohort (*p* = 0.0019). Progression-free survival was also significantly different for the three groups in the validation cohort (*p* = 0.0025). **Conclusions and Relevance:** An integrated machine learning model using SIRI, performance status, and smoking history was successfully developed and externally validated in oropharyngeal head and neck cancer patients.

## Simple Summary

We evaluated the prognostic value of the systemic inflammation response index (SIRI) in our retrospective cohort of patients with oropharyngeal head and neck cancer treated with curative-intent radiation therapy. By utilizing random survival forest and decision tree models, the incorporation of SIRI with performance status and smoking history significantly stratified overall survival into three distinct risk groups. Our model demonstrated both predictive accuracy and external validation, supporting the use of SIRI as a promising biomarker in oropharyngeal cancer.

## 1. Introduction

Standard-of-care treatment options for head and neck cancer of the oropharynx (HNC-OROP) include many possible combinations of systemic therapy, radiation, and surgery [1,2,3,4]. Treatment-related toxicities can be severe and frequently life-threatening [5]. Toxicities increase with increasing duration and number of modalities of therapy [6]. The human papilloma virus (HPV) status of the tumor and 10 or fewer pack years of smoking history defines three groups with distinct survival in HNC-OROP [7]. However attempts to de-intensify therapy in the best of these groupings have failed [8,9,10,11]. Therefore, better prognostication of survival outcomes is needed to optimize treatment selection to better match expected outcomes and toxicities.

Previously, we developed a prediction model using a machine learning approach for HNC-OROP patients’ survival based on 25 variables including those thought to reflect baseline inflammation. Though the total number of variables made clinical implementation challenging, this model had a high discriminative power (hazard ratio of 7.41, *p* < 0.0001). We noted that neutrophil percentages were negatively correlated with the lymphocyte percentage [12].

A higher neutrophil-to-lymphocyte ratio and high monocyte values are markers of worse HNC-OROP survival [13,14]. The previously defined systemic inflammatory or inflammation response index (SIRI), calculated as [(neutrophils x monocytes)/lymphocytes], accounts for such changes. Elevated SIRI has been implicated as prognostic factor for survival in a variety of malignancies [15,16,17,18,19], including head and neck cancer [20,21,22,23,24].

Although p16 is an established prognostic marker, it represents a relatively inflexible stratification system and does not fully capture the heterogeneity in outcomes [25]. Our model is designed for prognostic purposes, aiming to improve risk stratification beyond p16 status by integrating additional clinical and molecular features.

We hypothesized that the use of SIRI may produce a more parsimonious model of HNC-OROP outcomes. To test this hypothesis, we examined if the inclusion of SIRI in a machine learning model would produce a more clinically usable prognostic model for HNC-OROP survival outcomes.

## 2. Methods

Our study was performed under a protocol (EDR 103707) approved by Roswell Park Comprehensive Cancer Center institutional review board. The Strengthening the Reporting of Observational Studies in Epidemiology (STROBE) reporting guideline was reviewed, and our study follows the guideline.

Our retrospective database included all patients with primary HNC-OROP who underwent curative-intent definitive or post-operative radiation therapy at the Roswell Park Comprehensive Cancer Center between January 2013 and April 2024. Patients were excluded if they were diagnosed with metastatic cancer.

The machine learning cohort included 568 of these patients. All patients received a definitive radiation dose as appropriate based on the NCCN guideline (definitive patients received 70 Gy in 33–35 fractions and adjuvant patients received 60–66 Gy in 30 to 33 fractions per the treating physician’s discretion) [1].

### 2.1. Machine Learning Models

Random survival forests (RSFs) are an extension of RFs designed for analyzing time-to-event data, utilizing tree-based ensemble machine learning methods. In comparison to linear models, the RF model generally provides better prediction performance due to its ability to handle nonlinear relationships and complex interactions among predictors. For a new patient, the RSF estimates the survival probability at any given time point (survival function) following treatment, along with the cumulative hazard function. Variable importance is employed to assess the contribution of each independent variable to the model’s predictions. In this work, we evaluated two models: Model 1, a more complex, previously developed (full) model [12], and Model 2, a simplified (reduced) model. Model 1 contained 13 variables and a “host factor score” calculated by principal component analysis of the pre-treatment complete blood count. Model 2 took the top 4 features of the full model (KPS, host factor score, BMI, smoking) but replaced the host factor score with SIRI. The analyses were conducted using R 4.3.2 and R packages randomForestSRC [26], with 1000 trees and default settings.

### 2.2. Performance Metrics

The model’s prediction performance for overall survival (OS) was assessed using the concordance index (C-index). The C-index is an extension of the area under the curve (AUC) that accounts for censored data. It is calculated as the proportion of concordant pairs out of the total evaluable pairs. A pair is considered concordant if the subject with the higher predicted survival probability also has a longer survival time. We also assessed the model’s ability to predict patient survival at 1 to 5 years, using time-dependent receiver operating characteristic (ROC) curves.

### 2.3. Modeling Strategy

We primarily followed the modeling strategy as in Yu et al. [12]. Specifically, before any steps of model training, the cohort was randomly split into a training/validation set with 70% of subjects and a test set with 30% of subjects. Missing values were imputed using random forest imputation (300 trees and 5 iterations), which was performed without involving the outcome variables and strictly within the training, validation, and test cohorts. Model 1 uses a standard principal component analysis (PCA) for dimension reduction in the standardized host factors. The PCA was performed only within the training/validation set. For details, please see [12]. No test data set was used in any model training steps, including pre-processing or unsupervised learning by PCA. As the RSF requires minimal tuning of hyperparameters, we used the default settings with 1000 trees.

### 2.4. Model Interpretation

The permutation variable importance (VIMP) was utilized to evaluate the impact of each independent variable on the model’s predictions. VIMP quantifies the drop in prediction performance (C-index) of the forest ensemble when a variable is randomly shuffled. A high positive VIMP value signifies that permuting this variable notably diminishes the model’s accuracy, highlighting it as a potentially influential predictor.

### 2.5. Construction of a Robust Decision Tree

While the RSF model is effective, it is often regarded as a ‘black box,’ making it challenging to interpret and implement in practice. In contrast, decision trees naturally align with clinical decision-making processes and are highly user-friendly in clinical settings. However, they are prone to high instability during model training. To construct a more robust decision tree, we developed a customized approach by converting the continuous variables into categorical variables using potentially optimal cutoffs. To identify the cutoffs, we took advantage of the tree growing process in an RSF, where each decision rule was selected by maximizing the target statistic given the existing partition of the sample space. While this approach is greedy in nature, on average, the cutoffs should be centered around the optimal ones while conditional on other predictors. Under this assumption, all splitting rules involving the given continuous predictor were extracted from the RSF model. The distribution of the corresponding cutoffs was then estimated using a kernel density estimator. Next, the estimated density was compared against the distribution of the variable estimated using the training data. The cutoffs were selected as local maxima in the difference between the two densities. The cutoffs that resulted in minor groups with less than a proportion of 10% were excluded. After converting the continuous predictors into categorical variables, a decision tree was constructed using the standard approach implemented in R randomForestSRC package [27]. The maximal depth was set to 3 to avoid overly complicated rules, and the node size was set to 40.

### 2.6. Methods for External Validation

This validation was performed after obtaining Institutional Review Board approval at the Ohio State University Comprehensive Cancer Center (protocol 2024C0084). Its institutional database was queried for patients with non-metastatic oropharyngeal squamous cell carcinoma who received definitive radiation or chemoradiation between December 2011 and February 2024 with baseline SIRI available. All patients received a definitive radiation dose as appropriate based on the NCCN guideline (66 Gy in 30 fractions for select early-stage oropharyngeal cancer and 69.96 Gy in 33 fractions or 70 Gy in 35 fractions for all others per the treating physician’s discretion) [1]. All patients received intensity-modulated radiation therapy. Those with unknown smoking status, performance status, or SIRI at baseline were excluded for analysis.

Clinical variables of interest were extracted, including age, gender, race, smoking status, primary disease site, BMI, Eastern Cooperative Oncology Group (ECOG) performance status, p16 status, tumor staging based on the American Joint Committee on Cancer (AJCC) 7th edition, treatment types, chemotherapy, and SIRI. BMI is stratified as underweight (<18.5), normal (18.5–24.9), overweight (25–29.9), or obese (≥30). ECOG performance status was adjusted to follow the Karnofsky performance status scale.

The primary endpoint was overall survival (OS), defined as the time interval from diagnosis to death from any cause or last follow up. Another endpoint included progression-free survival (PFS). PFS was defined as the time interval from diagnosis to death from any cause, tumor progression, or last follow up.

### 2.7. Statistical Analysis

For risk stratification, the survival curves were estimated using Kaplan–Meier product limit estimators and compared using log-rank tests. The hazard ratios (HRs) were estimated based on Cox proportional hazards models, and the 95% confidence intervals (CIs) were reported.

### 2.8. Statistical Analysis for External Validation

Baseline characteristics were summarized using descriptive statistics. The validation of the model was performed after applying the cutoffs for the variables previously identified and stratifying the patient cohort into corresponding groups. Survival outcomes were analyzed using the Kaplan–Meier plot, log-rank test, and univariable Cox proportional hazards regression stratified by groups. Holm–Bonferroni correction was used for multiple comparisons when comparing among different groups. All *p* values were 2-sided, and those less than 0.05 were considered statistically significant. The validation analysis was performed using R version 4.5.1 (R Group for Statistical Computing).

## 3. Results

Patient characteristics are shown in Table 1. Median follow up was 33.1 months (interquartile range 13–71.3 months). Most patient characteristics are comparable. The external validation cohort from the Ohio State University tend to have higher SIRI scores and performance status. Our previous work identified Karnofsky Performance Status (KPS), body mass index (BMI), smoking status, and a composite host factor score as the top predictors of overall survival in head and neck cancer patients [12].

**Figure 1:**
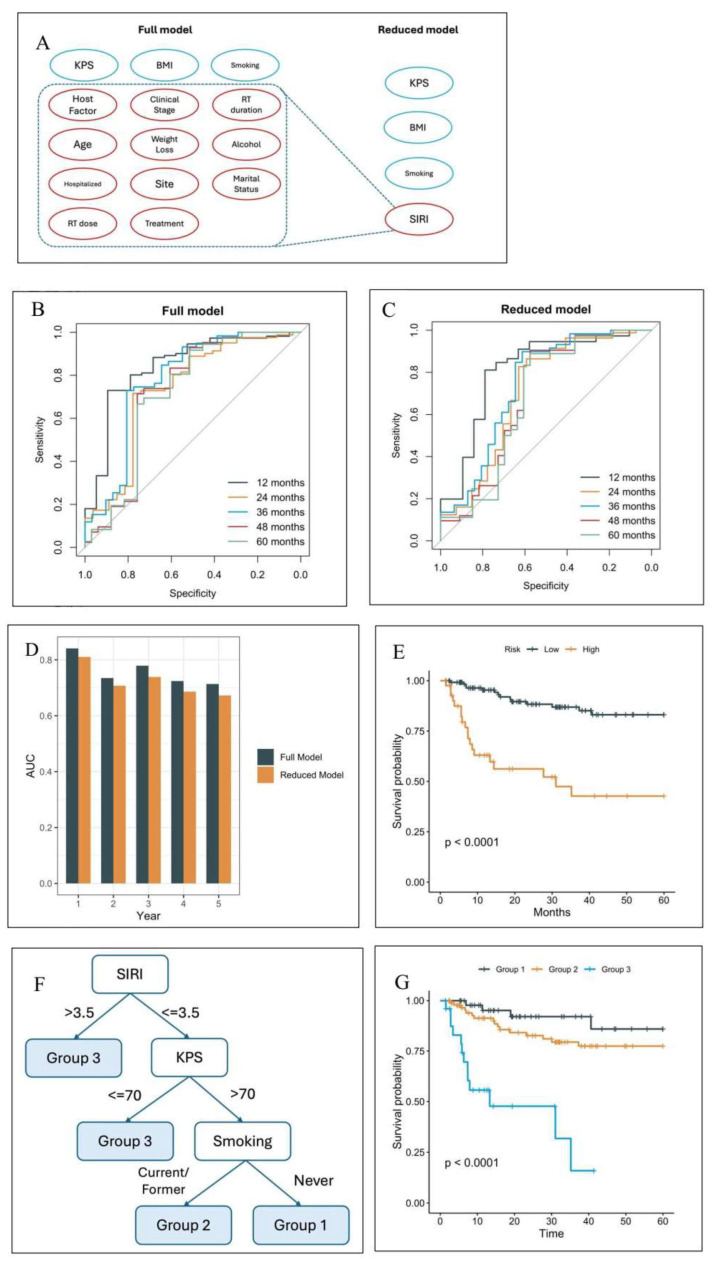
(**A**) In the current, reduced model, SIRI replaced several predictors from the prior, full model. (**B**) Receiver operating characteristic curves (ROCs) of the full model. (**C**) ROCs of the 4-feature model. (**D**) Comparison of the area under the curve (AUCs) at months 12–60. (**E**) The overall survival curves based on the risk stratification by the reduced model. (**F**) The decision tree of the parsimonious model partitions the cohort into four groups, where two high-risk groups are merged to form Group 3. (**G**) The overall survival curves of the three groups in the test set.

Figure 1A shows the variables utilized in the prior and current model with only four variables (SIRI, Karnofsky Performance Status (KPS), body mass index (BMI), and smoking status) in predicting overall survival. As shown in Figure 1B,C, the ROC curves for the prior (full) and current (reduced) model indicate that both models perform well in terms of discriminative power. Notably, the difference in AUCs between the two models remains small, with the maximum difference consistently below 0.05, as illustrated in Figure 1D. This suggests that the reduced model retains a comparable level of predictive accuracy, making it a viable alternative for situations where model complexity and ease of use are critical considerations.

To identify patients at the highest risk, the test cohort was further stratified based on the 75th percentile of the predicted risk scores derived from the simplified model. Figure 1E illustrates that patients in the high-risk group, as defined by this threshold, experience significantly worse overall survival compared to those in the low-risk group (hazard ratio 5.1, 95% confidence interval *p* < 0.0001). Therefore, the reduced model based on the four selected variables is sufficient to attain comparable performance versus the full model.

Random survival forest (RSF) models are effective but complex and unintuitive, making them difficult to implement in clinical practice. In contrast, decision trees are simple, intuitive, and naturally aligned with clinical decision-making. However, they are prone to high instability during model training. To construct a more robust decision tree, prior to the tree growing process, we identified the potentially optimal cutoffs as follows: KPS = 70, BMI = 25 and 30, and SIRI = 3.5. Stratification by these four variables created six risk groups. However, the survival curves of these groups clustered effectively as three groups (Figures S1–S3). Therefore, the reduced model was adjusted, and BMI was removed as a variable to create a further simplified, parsimonious model. This parsimonious model decision tree partitions the cohort into three groups (Figure 1F). The C-index of the models were 0.758 (RSF full), 0.725 (RSF reduced), 0.709 (decision tree reduced), and 0.702 (decision tree parsimonious). The parsimonious model was chosen for further study. Analysis of the internal validation cohort shows significant differences in overall survival among the three risk groups (Figure 1G, *p* < 0.0001). The calibration plot shows excellent agreement between predicted and observed 2-year survival probability in the internal test set (Figure S4A). The model was subsequently recalibrated. Additionally, the predicted risk groups showed excellent separation within HPV+, HPV−, and late-stage patients (Figure S5).

**Figure 2:**
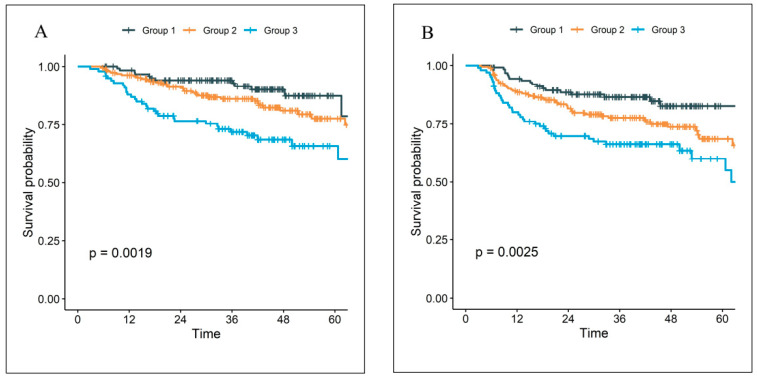
The Kaplan–Meier curves of (**A**) overall survival, (**B**) progression-free survival for the external validation cohort.

The external validation cohort of the parsimonious, 3-variable model used at The Ohio State University consisted of 421 patients with 85.0% men and a median age of 61 years [interquartile range 55–68]). Most patients were either former [49.4%] or current [14.5%] smokers who received definitive chemoradiation [94.8%]. The median follow up was 43.2 months (95% confidence interval [CI] 41.9–45.4). Kaplan–Meier plots are shown in Figure 2. There was a statistically significant difference among groups for OS (5-year OS: 87.5% for Group 1, 77.7% for Group 2, 65.8% for Group 3; *p* = 0.0019) and PFS (5-year PFS: 82.7% for Group 1, 68.6% for Group 2, 60.0% for Group 3; *p* = 0.0025). With three comparisons (Group 1 vs. 2, 1 vs. 3, and 2 vs. 3) and a Holm–Bonferroni correction, all comparisons remained statistically significant for OS, except for Group 2 vs. 1 (Group 2 vs. 1: hazard ratio [HR] 1.67, 95% CI 0.91–3.09, *p* = 0.10; Group 3 vs. 1: HR 2.87, 95% CI 1.53–5.38, *p* = 0.001; Group 3 vs. 2: HR 1.71, 95% CI 1.08–2.73, *p* = 0.02). For PFS, all comparisons were statistically significant, except for Group 3 vs. 2 (Group 2 vs. 1: HR 1.83, 95% CI 1.08–3.09, *p* = 0.02; Group 3 vs. 1: HR 2.60, 95% CI 1.49–4.52, *p* < 0.001; Group 3 vs. 2: HR 1.42, 95% CI 0.93–2.15, *p* = 0.10). However, the model systematically underestimates the survival probability in the external cohort (Figure S4B). In addition, the predicted risk groups did not show as good separation among the subgroups as in the internal test set (Figure S6). This may be explained by the difference in the patient populations, such as in SIRI scores and KPS (Table 1). The higher overall level of SIRI in the external cohort may result in an over-estimation of the risk in general (Figure S4C).

We also evaluated the robustness of the selected cutoffs based on randomly subsampled training data. The result suggests the cutoff selection based on our method is very stable (Figures S7–S9).

## 4. Discussion

Our parsimonious model, developed and validated on separate internal and external cohorts, defined three HNC-OROP groups with distinct overall and progression-free survivals using only three (SIRI, performance status, and smoking history) variables. The three main findings are that (1) higher SIRI (cut-off of 3.5) portended the worst prognosis regardless of other variables, (2) patients with low SIRI but poor performance status were also in the lowest-performing group, and (3) among patients with low SIRI and high performance, survival depended upon smoking history.

These findings concur with those of Valero et al., who studied 23 clinical and genomic variables that could predict PFS after immunotherapy in recurrent or relapsed HNC with 52% oropharynx patients. In their machine learning model, smoking, SIRI, and performance status were the first, second, and sixth most important out of 23 total variables including several genomic markers [28].

Machine learning models with a great many variables can closely approximate survival, but, due to complexity, are virtually impossible to deploy in the clinic. In our analysis, the full and reduced RSF models had a C-index of 0.758 and 0.725, respectively. The reduced and parsimonious decision tree models had a lower C-index of 0.709 to 0.702. This relatively minor penalty in C-index was certainly worth the increased simplicity.

Similarly, Valero et al. also produced a model simplification from 23 to 3 variables (SIRI, smoking status, and tumor mutational burden.) As in our analysis, higher SIRI portended the worst survival regardless of other variables. Also, the best survival was in non-smokers [28].

The HPV status of the tumor and 10 or fewer pack years of smoking history are widely used to define three groups with distinct survival in HNC-OROP [7]. Notably, HPV status was not chosen by the machine learning model as a variable in the full model, though HPV status and 10 or fewer pack years of smoking history define three groups with distinct survival in HNC-OROP in our cohort [29]. The fact that HPV status was unknown in 18 percent of our cohort offers one explanation. Second, there were only 14 HPV negative patients with fewer than 10 pack years of smoking; these patients had excellent survival and this may have impacted the initial modeling The NRG-HN005 phase II trial tested dose reduction in radiation therapy with cisplatin or immunotherapy against the standard RTOG 1016 regimen in patients with HPV-positive, non-smoking-associated oropharyngeal cancer. Both experimental arms failed to demonstrate non-inferiority in progression-free survival compared to standard therapy, which achieved an exceptional 98% two-year PFS, ultimately leading to the discontinuation of plans for a phase III trial [11].

Outcomes of HNC-OROP are worsened by a history of smoking [7,30]. We previously found that former smokers are distinct from current smokers and have similar survival to non-smokers [29]. However, the model grouped current and former smokers. Additionally, though eventually dropped from the parsimonious model, the selected cut-points for BMI are around 25 and 30; these are the standard thresholds for being overweight and obese. Interestingly, these are not the thresholds we found when we previously studied BMI [31] as an independent variable. The concordance of our machine learning-defined cut-points and standard threshold for BMI and discordance with our own previously published thresholds demonstrate the validity of our method and the independence of the machine learning process from external influence.

The development and validation of the parsimonious 3-variable decision tree have multiple implications for improved treatment selection in HNC-OROP. Current guidelines offer a multitude of standard-of-care methods to both escalate (addition of immunotherapy, induction chemotherapy, surgery, novel therapies) and de-escalate (reduced radiation dose or duration) the intensity and duration of curative therapy [1]. However, perhaps due to improper risk stratification, none of these alternatives have shown an overall survival benefit.

Better prognostication of survival outcomes may allow more informed choices about treatment intensity to better match expected outcomes and toxicities [32,33,34]. Moreover, future studies may attempt to modulate SIRI pre-treatment to improve outcomes.

Our retrospective study has inherent limitations. Regarding the machine learning model, we attempted to ameliorate these limitations by using an external validation cohort. The model was trained and internally validated using a population of patients with a primary tumor involving the oropharynx. The utility of this model in non-oropharyngeal patients needs further study.

In addition, the distribution of Karnofsky Performance Status (KPS) differed between cohorts, with the external cohort exhibiting a higher proportion of patients with higher KPS scores. The KPS imbalance may partially explain the observed differences in overall survival between cohorts and could affect model transportability, as the model was primarily trained on patients with relatively lower functional status. This suggests that while the model demonstrates good discrimination across cohorts, its calibration and absolute risk estimates may be less accurate in populations with systematically higher KPS. Future external validations in more diverse patient groups will be important to further assess and potentially recalibrate the model for broader clinical use. Future research utilizing SIRI-based risk stratification promises to impact clinical decision-making and personalize treatment for oropharyngeal head and neck cancer patients. Ultimately, SIRI-driven models could potentially minimize patient morbidity by tailoring treatment to specific patient populations.

## 5. Conclusions

A machine learning model including only three readily available pre-treatment factors (SIRI, performance status, and smoking history) is validated and defines three groups with distinct survival outcomes for oropharyngeal head and neck cancer. Our model demonstrated both predictive accuracy and external validation, supporting the use of SIRI as a promising biomarker in oropharyngeal cancer.

## Figures and Tables

**Table 1:** Patient characteristics.

	Roswell Park Machine Learning Cohort		The Ohio State External Validation Cohort	
**N**	568		421	
	N or Median	% or IQR	N or Median	% or IQR
**Groups**				
1			125	29.7%
2			195	46.3%
3			101	24.0%
**SIRI**	1.34	0.88–2.19	1.99	1.27–3.24
**Age**	61	54–67	61	55–68
**Gender**				
Female	104	18%	63	15.0%
Male	464	82%	358	85.0%
**Race**				
Caucasian	535	94%	390	92.6%
Other	33	6%	31	7.4%
Unknown				
**Smoking History**				
Never	174	31%	152	36.1%
Former	306	54%	208	49.4%
Current	88	15%	61	14.5%
**Body Mass Index**				
Normal	124	22%	83	19.7%
Underweight	11	1.9%	9	2.1%
Overweight	221	39%	134	31.8%
Obese	201	35%	195	46.3%
Not available	11	1.9%	83	19.7%
**Karnofsky Performance Status**				
100	151	27%	275	65.3%
80–90	363	64%	128	30.4%
70–40	54	9%	18	4.3%
<40				
Not available				
**p16 Status**				
Negative	75	13%	43	10.2%
Positive	388	68%	378	89.8%
Unknown	118	18%		
**Tumor Stage**				
1–2	337	59%	288	68.4%
3–4	229	40%	133	31.6%
Unknown	2	0.4%		
**Nodal Stage**				
0–1	210	37%	87	20.7%
2–3	353	62%	334	79.3%
Unknown	5	0.9%		
**Treatment type**				
Definitive chemoradiation	427	75.2%	399	94.8%
Radiation alone	30	5.3%	22	5.2%
Induction chemotherapy, then definitive chemoradiation	51	9%		
Surgery then adjuvant (chemo)radiation	60	10.5%		
Other				
**Chemotherapy**				
None or other chemo	139	24.5%	179	42.5%
Cisplatin	429	75.5%	242	57.5%

## Data Availability

The data underlying this article cannot be shared publicly, to respect the privacy of the individuals that participated in the study. The data are available from the corresponding author upon reasonable request.

## Supplementary Materials

The following supporting information can be downloaded at https://www.mdpi.com/article/10.3390/cancers17233820/s1, Figure S1: Cutoff selection of three continuous variables; Figure S2: Variable importance of the reduced RSF model; Figure S3: (A) The decision tree with 4 variables stratifies the cohort into 6 groups. (B) Overall survival for the 6 risk groups clusters effectively as 3 groups. (C) The progression free survival curves of these 6 groups clustered effectively as 3 groups. Figure S4: Calibration of the 2-year survival probability in the internal test cohort (A) and the external validation cohort (B). A linear calibration model was fitted on the log(−log) scale, yielding an intercept of −1.288 and a slope of 0.497. The model was subsequently recalibrated, and the recalibrated survival probabilities were compared with the original model predictions (C). Figure S5: Kaplan–Meier curves of three risk groups within HPV+ (A), HPV− (B), Stage 1–2 (C) and Stage 3–4 (D) patients in Roswell Park test cohort. Figure S6: Kaplan–Meier curves of three risk groups within HPV+ (A), HPV− (B), Stage 1–2 (C) and Stage 3–4 (D) patients in OSU external validation cohort. Figure S7: Density plot for selecting the optimal cutoff of SIRI in 12 randomly subsampled training data. Figure S8: Density plot for selecting the optimal cutoff of KPS in 12 randomly subsampled training data. Red vertical line indicates selected cutoff in the main analysis. Figure S9: Density plot for selecting the optimal cutoff of BMI in 12 randomly subsampled training data.
