# Supplementary material for: Development and External Validation of Integrated Machine Learning-Based Prognostic Model in Oropharyngeal Head and Neck Cancer Using the Systemic Inflammatory Response Index
Source: Cancers (Basel). 2025 Nov 28;17(23):3820. doi: 10.3390/cancers17233820 (PMC12691270; doi:10.3390/cancers17233820)
Supplement: Supplementary file 1 [file cancers-17-03820-s001.zip › cancers-3930028-supplementary.pdf]

cancers

MDPI

Supplementary Materials: Development and External Validation of Integrated Machine Learning-Based Prognostic Model in Oropharyngeal Head and Neck Cancer Using the Systemic Inflammatory Response Index

Anurag K. Singh, Sung Jun Ma, Dukagjin Blakaj, Simeng Zhu, Neil D. Almeida, Andrew Koempel, Guangwei Yuan, Grace Wang, Kimberly Wooten, Vishal Gupta, Ryan McSpadden, Moni A. Kuriakose, Michael R. Markiewicz, Song Yao, Wesley L. Hicks, Jr., Mukund Seshadri, Elizabeth A. Repasky, Elizabeth G. Bouchard, Mark K. Farrugia and Han Yu

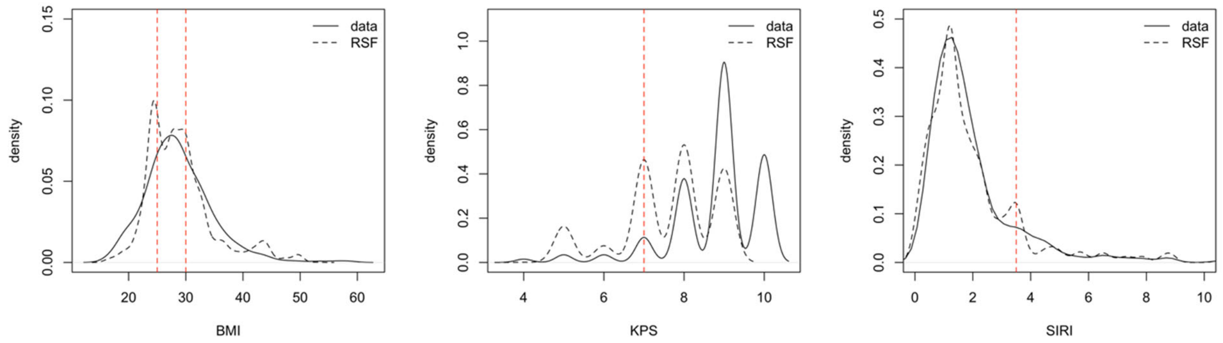

Figure S1. Cutoff selection of three continuous variables. The distribution of the RSF cutoffs was estimated using a kernel density estimator (dashed). The estimated density was compared against the distribution of the variable estimated using the training data (solid). The cutoffs (red) were selected as local maxima in the difference between the two densities. The extreme cutoffs that would result in minor groups with less than a proportion of 10% were excluded.

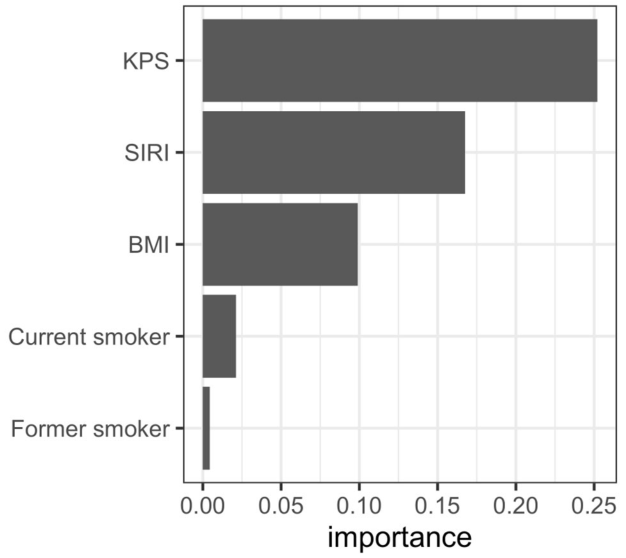

Figure S2. Variable importance of the reduced RSF model.

 https://doi.org/10.3390/cancers17233820

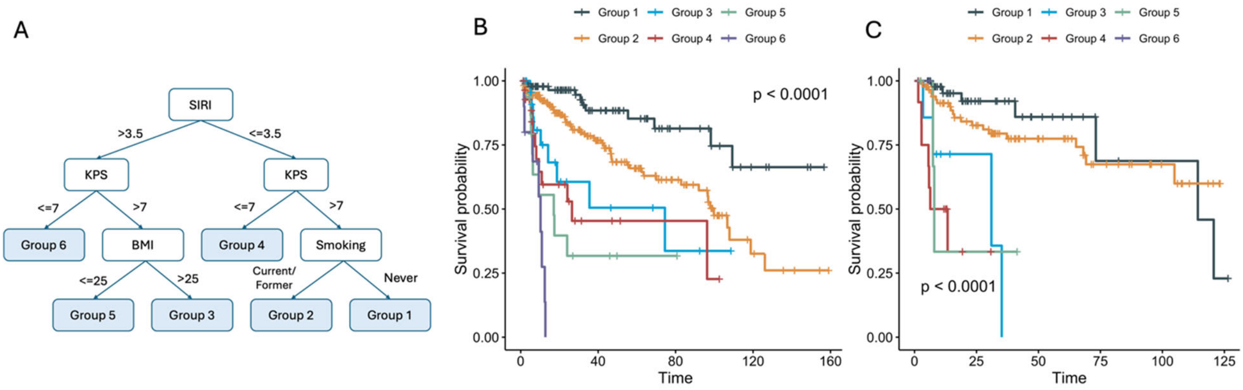

Figure S3. (A) The decision tree with 4 variables stratifies the cohort into 6 groups. (B) Overall survival for the 6 risk groups clusters effectively as 3 groups. (C) The progression free survival curves of these 6 groups clustered effectively as 3 groups.

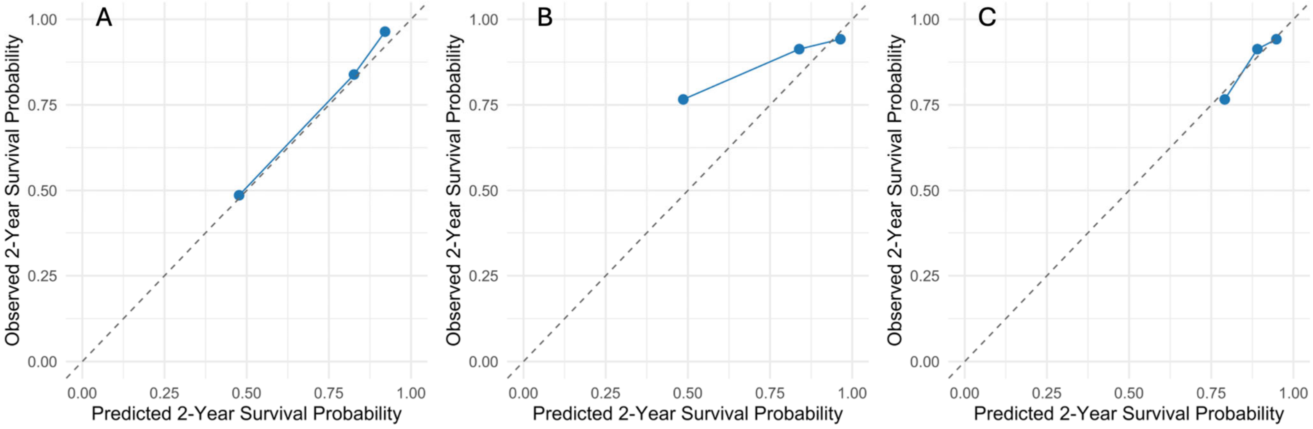

Figure S4. Calibration of the 2-year survival probability in the internal test cohort (A) and the external validation cohort (B). A linear calibration model was fitted on the log(–log) scale, yielding an intercept of –1.288 and a slope of 0.497. The model was subsequently recalibrated, and the recalibrated survival probabilities were compared with the original model predictions (C).

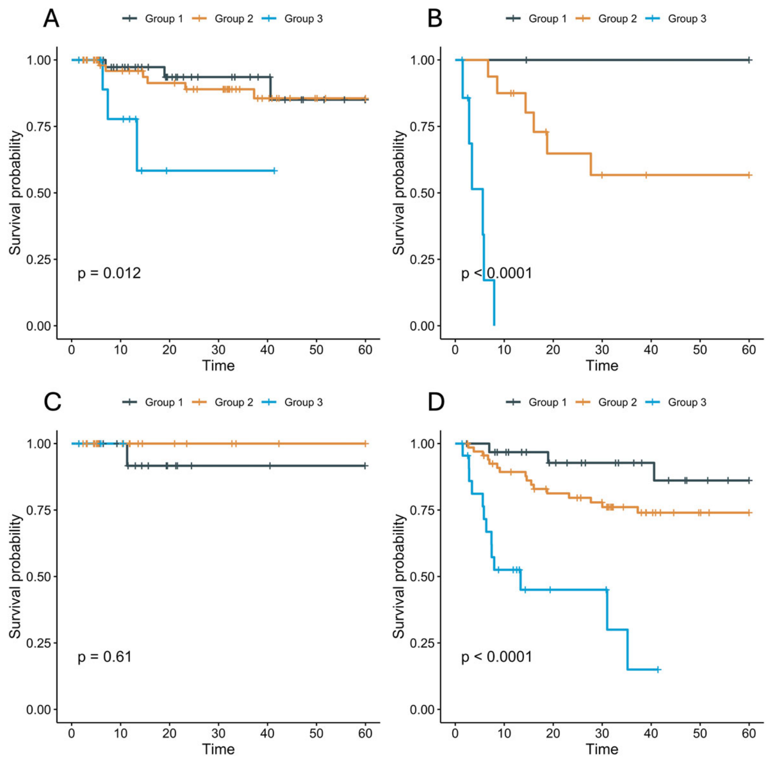

Figure S5. Kaplan-Meier curves of three risk groups within HPV+ (A), HPV-(B), Stage 1-2 (C) and Stage 3-4 (D) patients in Roswell Park test cohort.

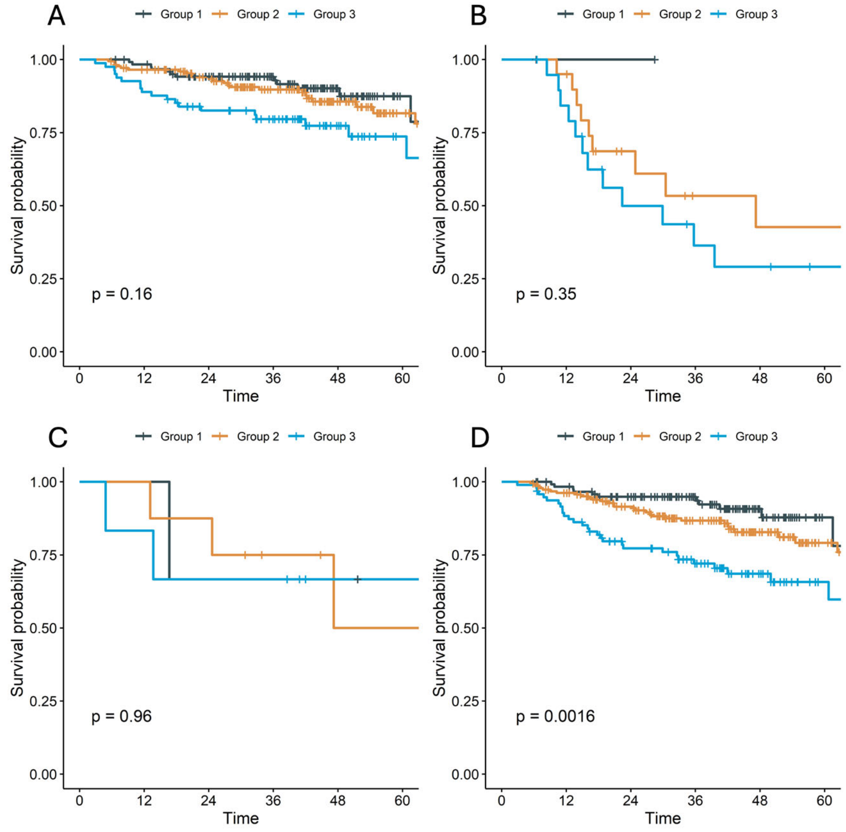

Figure S6. Kaplan-Meier curves of three risk groups within HPV+ (A), HPV-(B), Stage 1-2 (C) and Stage 3-4 (D) patients in OSU external validation cohort.

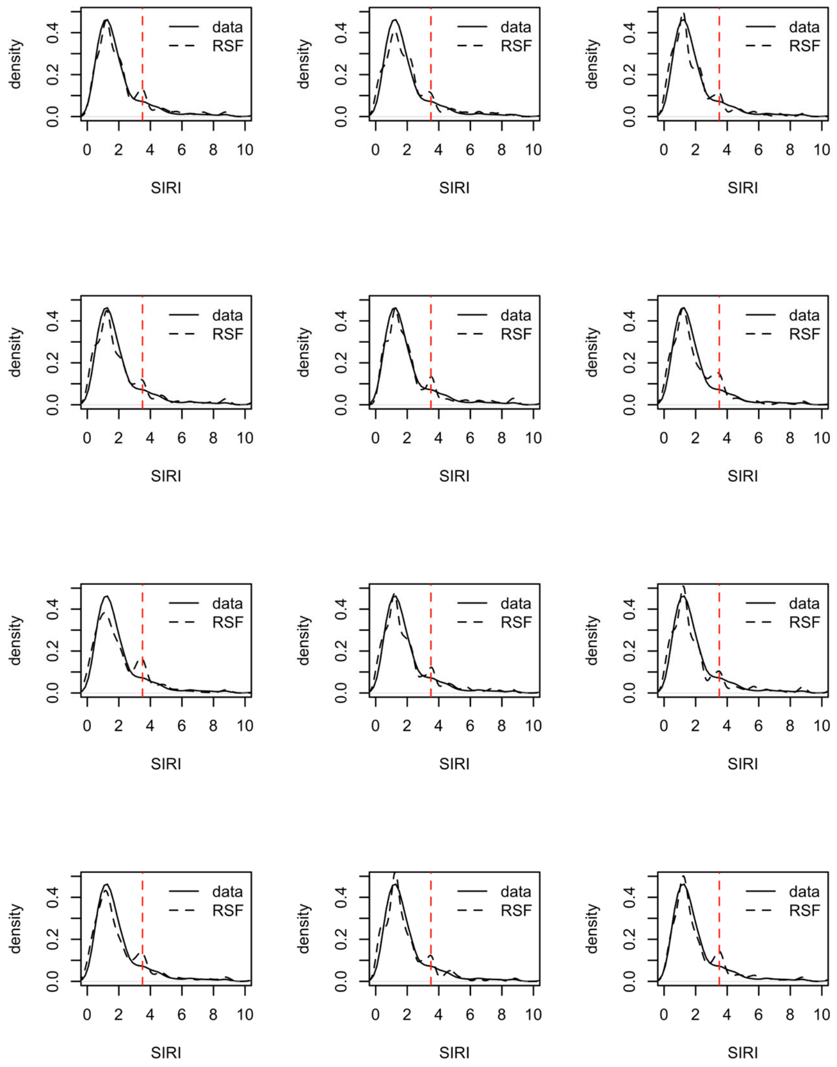

Figure S7. Density plot for selecting the optimal cutoff of SIRI in 12 randomly subsampled training data. Red vertical line indicates selected cutoff in the main analysis.

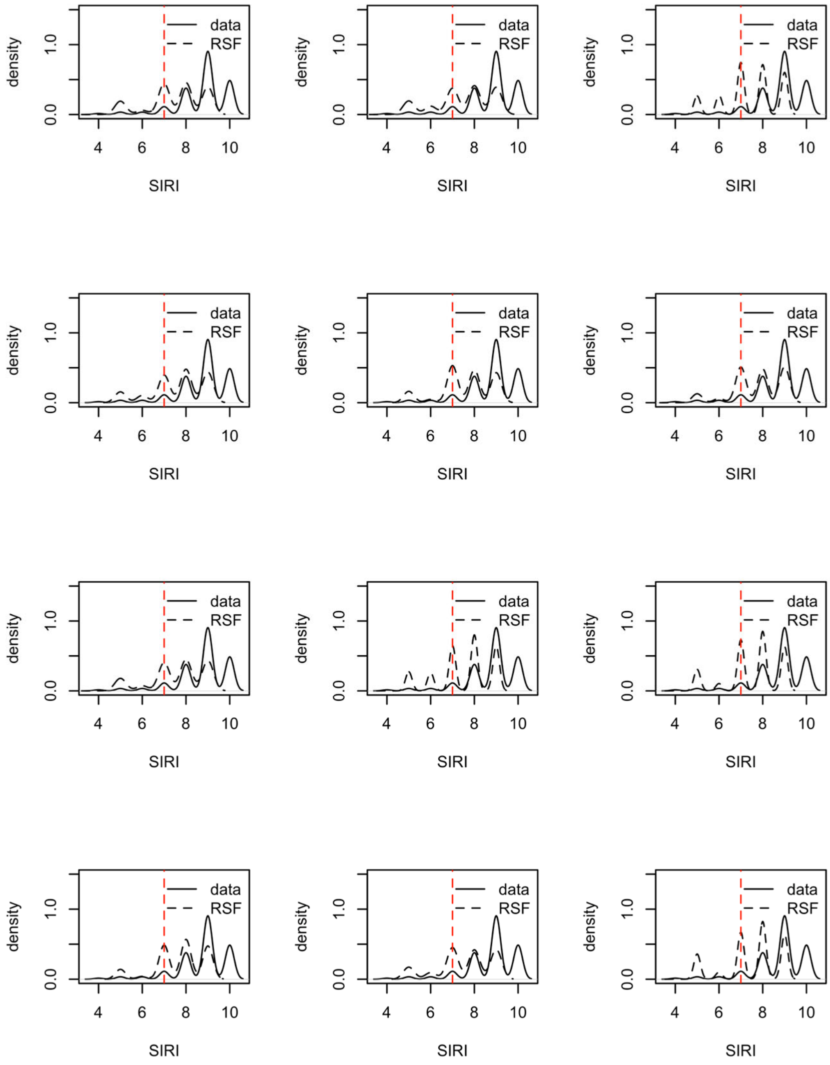

Figure S8. Density plot for selecting the optimal cutoff of KPS in 12 randomly subsampled training data. Red vertical line indicates selected cutoff in the main analysis.

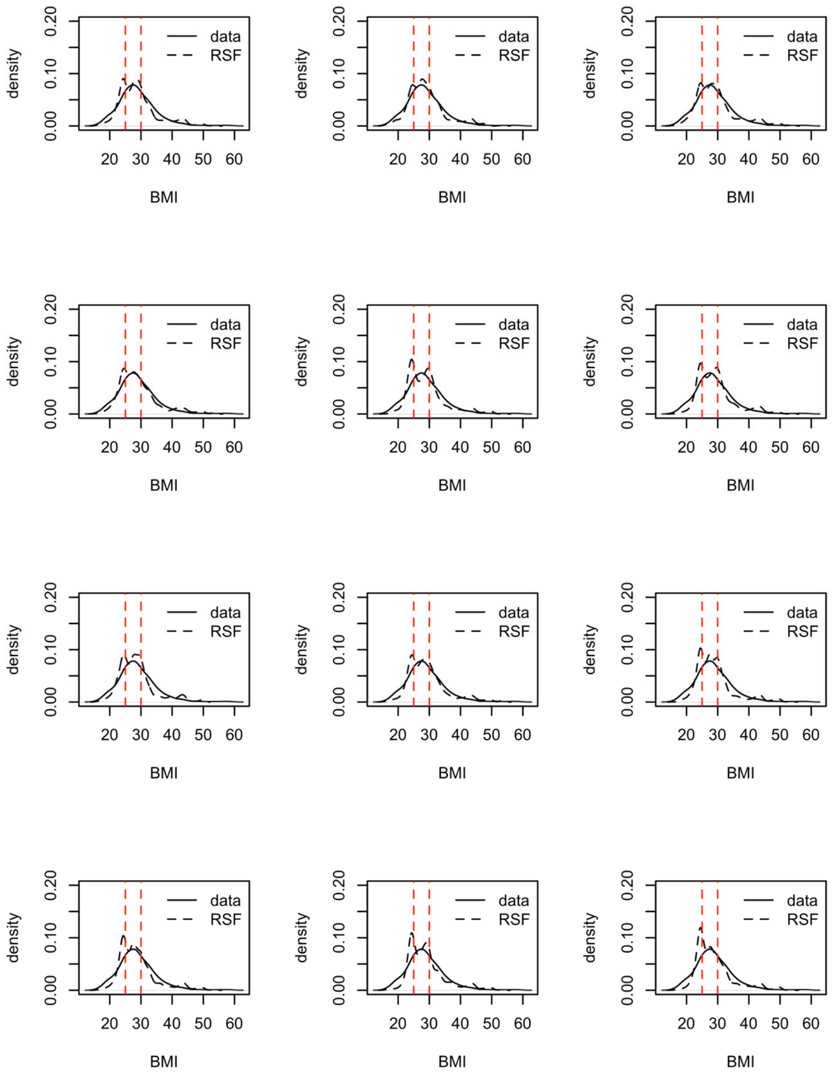

Figure S9. Density plot for selecting the optimal cutoff of BMI in 12 randomly subsampled training data. Red vertical line indicates selected cutoff in the main analysis.
